# On the Treatment of Chronic Catarrh of the Bladder, and Some Forms of Acute Cystitis

**Published:** 1879-02

**Authors:** Theodore Deecke

**Affiliations:** Special Pathologist, New York State Lunatic Asylum, Utica, N. Y.


BUFFALO

Medical and Surgical Journal.

VOL. XVIII. FEBRUARY, 1879. No. 7.

### Original Communications.

:o:

*ART. I.—On the Treatment of Chronic Catarrh of the Bladder, and of some forms of Acute Cystitis.* By THEODORE DEECKE, Special pathologist, New York State Lunatic Asylum, Utica, N. Y.

Acute cystitis and chronic catarrh of the bladder or cystorrhœa are not to be considered as stages of the same disease. Acute cystitis may develop, when neglected, a chronic catarrh, yet more frequently the latter makes its appearance as a characteristic chronic affection from the beginning. It is not the aim in the following pages to give a history and a description of the diseases which have been so well described in all their particulars in the "Treatise on the Diseases of the Bladder," by S. D. GROSS, 1851, and by other authors in this country and abroad.

As the most important direct cause in the majority, if not in all cases of chronic catarrh of the bladder, we must denominate the involuntary retention of urine in the bladder, a condition which may be brought about by various local or general disorders. Such are, strictures of the urethra; hypertrophy of the prostate; paralysis from atrophy, or from morbid affections of the central nervous system; foreign bodies, which produce a decomposition of the urine in the bladder:—air and germs of microscopic forms of life.

The chronic catarrah usually developes in a slow, gradual and insidious manner, and the inflammation which accompanies the same is, at least in the beginning, almost always of a very mild grade; while in acute cytitis the symptoms of a severe and painful local inflammation, in early stages, point toward the site and the nature of the affection. In the course of the latter disease, however, morbid conditions may develop to some degree similar to those of the chronic catarrh, which may require the same interference in the way of medical treatment. This, for instance, will be the case when, as frequently occurs, for some length of time, only an incomplete evacuation of the bladder can be performed. The involuntary retention of urine, therefore, and its consequences is a feature in both diseases, and this may justify a consideration of both from a common point of view.

It has long been known that in chronic catarrh of the bladder as well as in acute cystitis of some standing, the urine which is passed is in an altered condition. Aside from being mixed with blood, pus or mucus corpuscles and degenerated epithelium cells, it is generally more or less alkaline in its character, emits a peculiar ammoniacal, often offensive odor, is rapidly decomposed, inside the bladder as well as out of it, and precipitates, either fresh or after a shorts tanding, numerous crystals of the triple-phosphates and of urate of ammonia. A constant occurrence, furthermore, are micrococci or globular masses of bacteria (gliococci, Billroth), to the fermentative action of which the rapid decomposition of theurine must be ascribed.

It is evident, and has long been acknowledged, that the alteration and decomposition of the urine inside the bladder in these affections, especially its alkaline condition, the crystalline deposits, the growth of the bacteria, etc., that they all must act as steadily irritating agents upon the already inflamed mucous membrane of the organ. An important part of the treatment, therefore, for the last ten years has been directed with more or less success against these urgent and uncontrollable influences. L. Wilcox* recommended the use of sulphite of soda. The urine was fetid, alkaline, contained pus and was passed every fifteen minutes. After the internal use of the remedy the urine became normal and could soon be retained for hours. Mineral acids were administered in these cases without success. Dubrueil† made injections into the bladder of silicate of soda (solution of 1 per cent.) in a case of hypertrophy of the prostate and had good results in preventing a decomposition of the urine. Th. Clemens‡ injected the urine of healthy persons into the bladder of patients suffering from chronic inflammation. He ascribed the good effects of it to the favorable influence of a healthy urine upon the walls of the affected organ!!! and even he found a successor in H. S. Purdon§. Benzoic acid, first recommended by Ure, was administered by Robin and Gosselin‖, especially in cases of highly alkaline reaction of the urine. Benzoic acid is in the system transformed into hippuric acid and in place of carbonate of ammonia hippurate of ammonia is formed in the urine which is claimed to be less poisonous and less apt to form concretions than the ammonio-triple phosphates. Internal doses and injections of carbolic acid were first administered by Déclat¶ with remarkable effect. Braxton Hicks** recommended in acute stages injections of morphine; in later stages, nitrate of silver, tannin and chloride of iron. The first who studied the effect of salicylic acid, in doses of one to two grammes *pro die*, in cystitis with alkaline fermentation was P. Fürbringer††. According to his experience salicylic acid removes the causes and the products of the fermentation, but it does not arrest the formation of pus. Also injections of the acid have been tried by him without much success. The newest essay on the treatment of chronic catarrh of the bladder has been written by Max Schüller‡‡. Even the largest doses of salicylic acid had not the desired effect. Scühller puts the

*The use of sulphite of soda in chronic cystitis. Brit. Med. Jour., Sept. 19, 1868.

†Injections de silicate de soude dans la vessie contre l' etat ammoniacal des urines. Gaz. des Hopit., 1872, No. 136.

‡Ueber Heilung chronischer Blasenkrankheiten mittelst Einspritzungen, etc. Deutsche Klinik, 1873. No. 7.

§Note on the treatment of cystitis, Dublin Jour., October, 1873.

‖Traitement de la cystite ammoniacale par l' acide benzoide. Arch. gen. de Therap ,etc., 1874.

¶L'efficacite des injections d' acide phenique dans la vessie et de 'l administration interne du sirop d' acide phenique dans les cas de cystite avec. urines ammoniacales. Comp. rend. LXXVIII, No. 4, 1874.

**The local treatment of cystitis in women. Brit. Med. Jour., 1874, July 11.

††Berliner Klinische Wochenschrift, 1875, 19.

‡‡Ueber die Lokalbehandlung des chronischen Blasencatarrhes. Berlin, 1877.

most weight on the local treatment, and he distinguishes between remedies which prevent a decomposition of the urine and others which produce an effect upon the inflamed mucous membrane of the bladder. Of the first, carbolic acid had not in all cases the desired effect of producing an acid reaction of the urine. It is purely an antiseptic and gives good results in solutions of one to one and a half per cent. in higher stages of alkaline fermentation and in diphtheritic affections. Salicylic acid in solutions of 0.3—0.5 per cent, locally applied, gave better results than by internal administration. It is a good antiseptic and dissolves purulent deposits into a liquid emulsion which can be easily removed. It prevents an agglutination of the pus-corpuscles and acts favorably upon the catarrhal inflammation of the mucous membrane. It is less active against the glairy mucoid deposits. Hyper-manganate of potassa in solutions of 0.05 to 0.2 per cent. is a good antiseptic, yet only useful without pain in very weak solutions. There is no better medium for the purpose of dissolving the muco-purulent deposits than chloride of sodium in solutions of 5 per cent., by which a uniform emulsion is produced which can be completely evacuated. Nitrate of silver 0.5—1.0 per cent. and chloride of zinc 1. to 2.0 per cent. are recommended in ulcerative processes of the mucosa and copious formations of pus.

It is about two years ago that my attention was called to the almost constant occurrence of micrococci and gliococci in the urine of patients who suffered from chronic catarrh of the bladder or from those forms of acute cystitis which were accompanied by an excretion of urine of a neutral or alkaline reaction. This occurrence induced me to a series of experiments with different substances, prominently acids, for the purpose of studying their action upon the development and the growth of those organizations, and of exploring their influence upon the alteration of the liquid which served as their pabulum. The experiments were first made in open test tubes, and were repeated by excluding the air as much as possible. The pabulum was normal urine and urine of a patient who had been suffering a number of months from a mild chronic catarrh of the bladder and who had been treated, as he related, for "some form of Bright's disease of the kidneys," the general dread of all who experience some chronic disorder in the uropoëtic system. The tried substances were: silicate of soda; sulphite of soda; permanganate of potassa; sulphuric, nitric and muriatic acid; acetic, citric and tartaric acid; carbolic acid; salicylic acid; and lactic acid.

As the experiments were of a decided result in one direction it is of no interest and consequence here to enter in the narration of any details combined with them.

There is among all these substances only *one* the effect of which was found to be far beyond comparison with any of the others, and this is *lactic acid.* An addition of one per cent. of lactic acid prevents a decomposition and alkaline fermentation of normal urine and the development of micrococci and gliococci for a long period; in pathological urine it arrests their growth and multiplication almost instantly. It seems to be a specific poison for these microscopic forms of life.

As the antiseptic properties of lactic acid have been known for years, it is the more surprising that it has not been tried long ago in these special cases, since all remedies hitherto recommended, according to the experience of the latest expert, Dr. Max Schüller* of Greifswald "only met the one or the other demand with the desired effect..'

It may be remarked here at once that the antiseptic action of lactic acid, by local application, is by far not the only effectual property of this drug. Its dissolving power for catarrhal and diphtheritic exudations is likewise known, and should not be undervalued; it is superior to that of any other acid. It furthermore dissolves easily the ammonium compounds, the ammonio-triple-phosphates and the calcium phosphates. And, best of all, it permits also of an internal administration in a most agreeable form, as a kind of lemonade, or in the form of buttermilk or added to it. It will be discovered in the excreted urine, after a few good doses, a fact which was established by a number of careful analyses of the urine after internal administration of the acid. This generally occurs when three or four grammes of the drug have been consumed, an effect which will be accelerated by a

*l. c. page 50.

cotemporary use of buttermilk as a beverage. The latter property of course makes the remedy the more valuable, especially in all acute cases and those of a milder course, or where the condition of the patient or other circumstances are not favorable to a local treatment. As to the administration of the drug, the acid may be given in doses of 1.0 to 1.5 even to 2.0 grammes three times a day in sugar and water. Even the large doses administered in a diluted form or in a mild bitter tonic, when continued for a longer period, do not interfere with the digestion. A hypnotic influence has not been observed. For local treatment solution of 0.5 to 1.0 per cent. will suffice, and I recommend two injections at one session for the beginning, with the cotemporary internal use of the acid; and, not to repeat the injection sooner than absolutely necessary. In the majority of cases, even of long standing, only a few injections will be required.

As to the instruments for injection into the bladder, a simple silver catheter is preferred to those with a double tube; for a syringe I recommend a fountain syringe or syphon syringe (like the common nasal douches) with a rubber tube of about three feet in length. If the catheter is provided with a stop-cock at its base part, or with a short rubber tube closed by a clamp, it can be introduced filled with the injecting fluid and the connections can be made with the fountain tube in such a manner that all air is excluded inside of the apparatus which will be found of great convenience to the patient as well as to the attending physician. The manipulations are simple. The tumbler containing the injecting fluid, the temperature of which should be raised to about 100° F., is placed on the floor, the tubes are filled with the fluid and the connections are made with the catheter in position, which must be supported by the left hand or by the patient himself. The right hand then raises the tumbler from the floor, slowly, to the desired height. The bladder fills up by this arrangement without any inconvenience to the patient, or any sudden shock, and the pressure from the column of the injecting fluid can be entirely controlled and the bladder can be expanded ad libitum.

During about eighteen months twenty-one cases have been under treatment, of which a record was made, for the most part by practical physicians of my acquaintance. Among these there is only one case to be mentioned of a chronic catarrh of the bladder from a stricture in which the recovery remained doubtful because of an interruption in the treatment. In six acute cases (5 m., 1 f.) the cystitis had originated from an inflammatory condition of the urethra; in only one of these it was found necessary to have recourse to a twice repeated injection into the bladder. Five acute cases (3 m., 2 f.), developed without any known cause, recovered rapidly from an internal use of the acid. One case of a chronic catarrh from partial paralysis of the bladder required three double injections; one case of hypertrophy of the prostate four injections. From eight cases of chronic catarrh of long standing only in one the injection was repeated ten times, three recovered from the internal use only and the remaining four cases submitted to two, to three and four injections.

In conclusion it might be found of some interest that George Johnson (Lancet 2, 1876, page 847.) in affections of the bladder highly recommends a "milk diet."

